# Primary Cardiac Tumours: A Single-Center 41-Year Experience

**DOI:** 10.5402/2012/906109

**Published:** 2012-06-27

**Authors:** Christina Maria Steger, Thomas Hager, Elfriede Ruttmann

**Affiliations:** ^1^Department of Pathology, Innsbruck Medical University, Müllerstraße 44, 6020 Innsbruck, Austria; ^2^Department of Pathology, Ludwig-Maximilians-University Munich, Thalkirchner, Straße 36, 80337 Munich, Germany; ^3^Department of Pathology and Neuropathology, University of Duisburg-Essen, Hufelandstraße 55, 45147 Essen, Germany; ^4^Department of Cardiac Surgery, Innsbruck Medical University, Anichstraße 35, 6020 Innsbruck, Austria

## Abstract

Primary cardiac tumours are extremely rare with the most commonest being left atrial myxomas. In general, surgical resection is indicated, whenever the tumour formation is mobile and embolization can be suspected. Within 17280 patients receiving heart surgery at the Innsbruck Medical University, 78 patients (0.45%) underwent tumourectomy of primary cardiac tumours. The majority of patients (63) suffered from a left or right atrial myxoma, 12 showed a papillary fibroelastoma of the valves at echocardiographical or histological examination, 1 suffered from a hemangioma, 1 from a chemodectoma, and another one from a rhabdomyosarcoma. The mean age of cardiac tumour patients was 54.29 ± 13.28 years (ranging from 18 to 83 years). 67.95% of the patients were female and 32.05% were male. The majority of tumours were found incidentally; 97.44% of the patients showed no tumour recurrence.

## 1. Introduction

Primary cardiac tumours are extremely rare. They are found in only 0.01% of autopsies [1], metastatic tumours being nearly 100 times more common. Approximately 75% of primary cardiac tumours are benign.

Tumours of the heart may be classified broadly into three types: benign tumours and tumour-like lesions, malignant tumours, and pericardial tumours [2] (Tables 1 and 2).

Cardiac tumours can present a significant diagnostic challenge causing symptoms and signs that mimic other cardiac diseases. Main symptoms include breathlessness, fever, weight loss, syncope, haemoptysis, and sudden death.

Cardiac tumours may also cause embolization, arrhythmias (atrioventricular block and ventricular tachycardia), or obstruction of the outflow tracts. Diagnosis depends on a high index of suspicion and can almost always be made by echocardiography.

Differentiation of cardiac tumours from valvular vegetation and atrial thrombus is important, and usually the echocardiographic appearance of a myxoma or a papillary fibroelastoma is quite distinctive.

Benign tumours normally carry a good prognosis with normal life expectancy after resection. Patients who have had benign tumours resected are usually followed up with regular echocardiography and cardiological supervision. Malignant tumours such as sarcomas tend to have a poor outcome despite intervention, with a median survival from initial diagnosis of about 6 months. Occasional cases of survival due to complete resection do occur. Secondary malignancy affecting the heart has a grave outlook, although there is much that can be done to palliate the worst effects of the condition.

## 2. Material and Methods

All patients undergoing surgery due to primary cardiac tumours from January 1970 to December 2011 were included in this study. Our study focussed on preoperative symptoms, the occurrence of thrombembolic events, strokes or transient ischemic attacks, echocardiographic findings, histological diagnosis of excised specimens, and recurrence rate. Mean follow-up duration was 109.25 ± 113.91 months (ranging from 3 to 457 months).

## 3. Results

**Figure 1:**
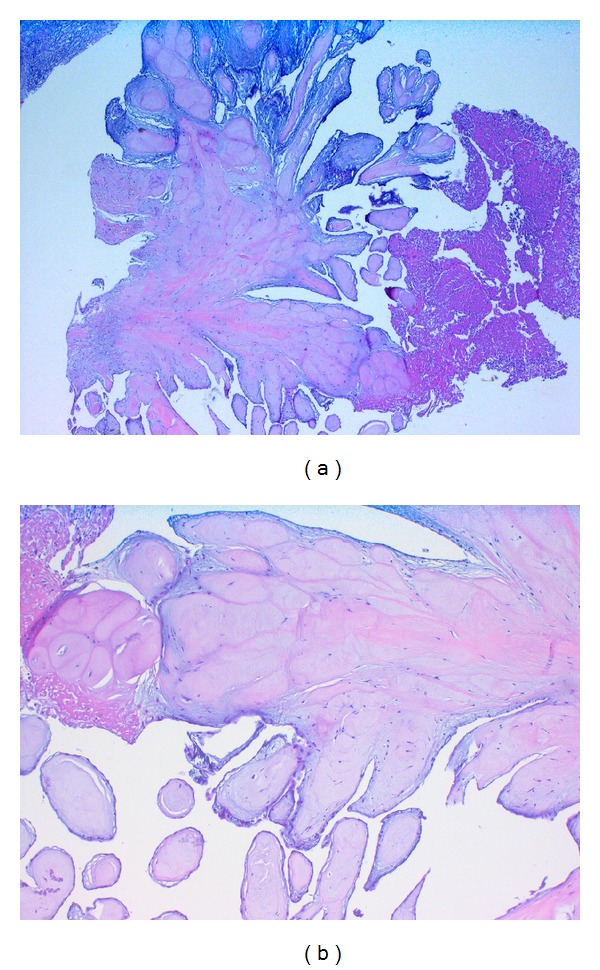
(a) and (b) Papillary fibroelastoma of the aortic valve with papillary configuration with an avascular connective tissue core lined by a single layer of endothelial cells (H&E: on the left side, original magnification ×100; on the right side, original magnification ×400).

**Figure 2:**
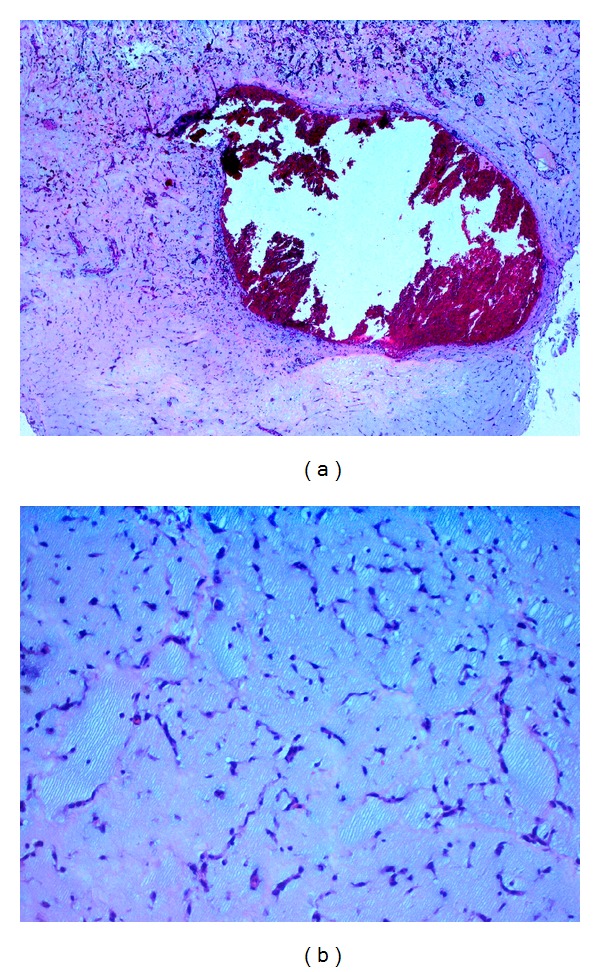
(a) Left atrial myxoma consisting of a mucoid stroma with spindle-shaped cells, with hemorrhage in the centrum of the myxoma (H&E staining, original magnification ×100); (b) High power view (H&E staining, original magnification ×400).

**Figure 3:**
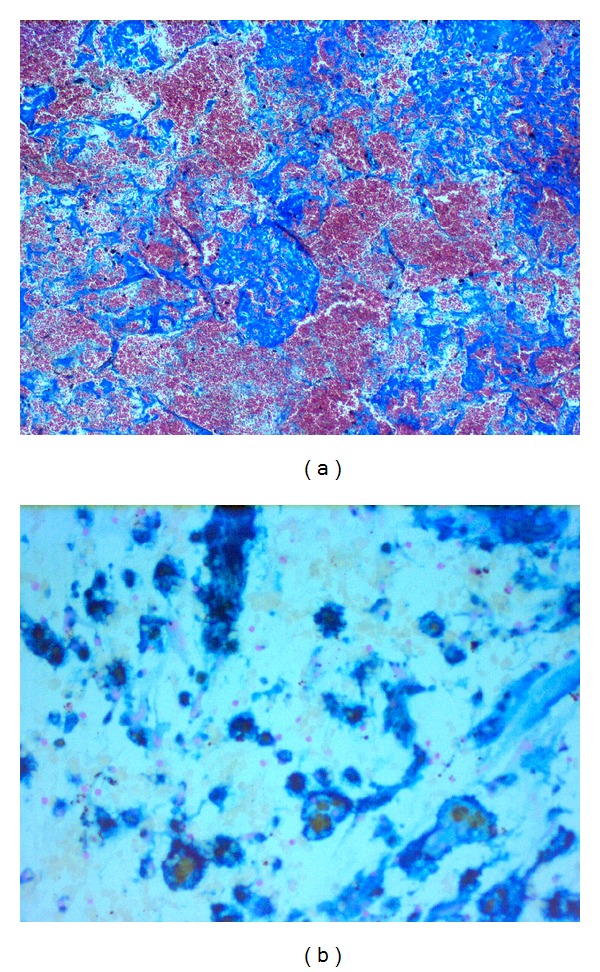
(a) CAB (Chromotrope Aniline blue) staining of myxoma tissue colouring fibrous tissue blue and vascularized areas red (CAB staining, original magnification ×100). (b) Iron stain of macrophages colouring iron yellowish (Iron stain, original magnification ×600).

**Figure 4:**
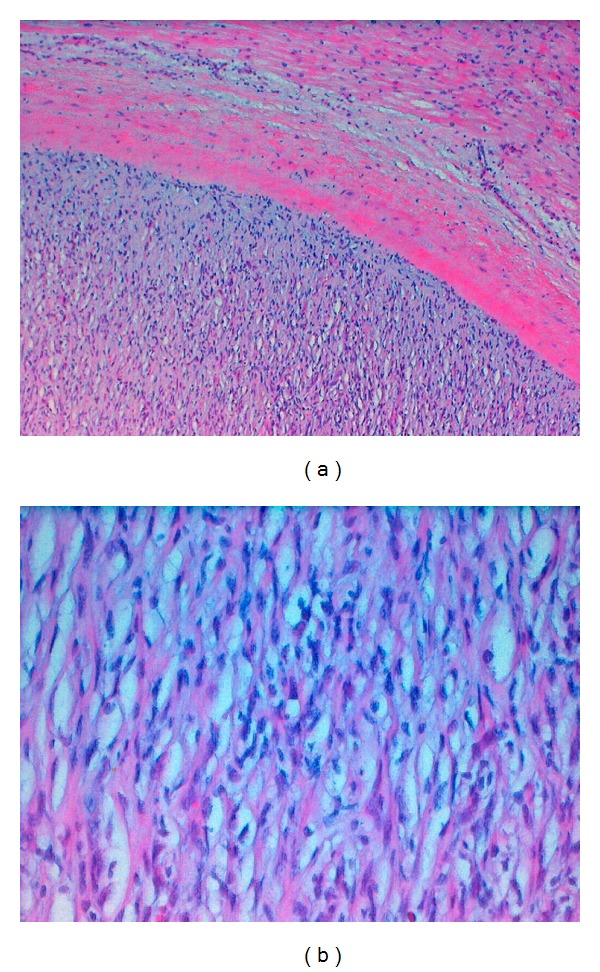
(a) Rhabdomyosarcoma surrounded by a fibrous capsule and myocardial tissue (H&E staining, original magnification ×100); (b) high power image of rhabdomyosarcoma cells (H&E staining, original magnification ×400).

**Figure 5:**
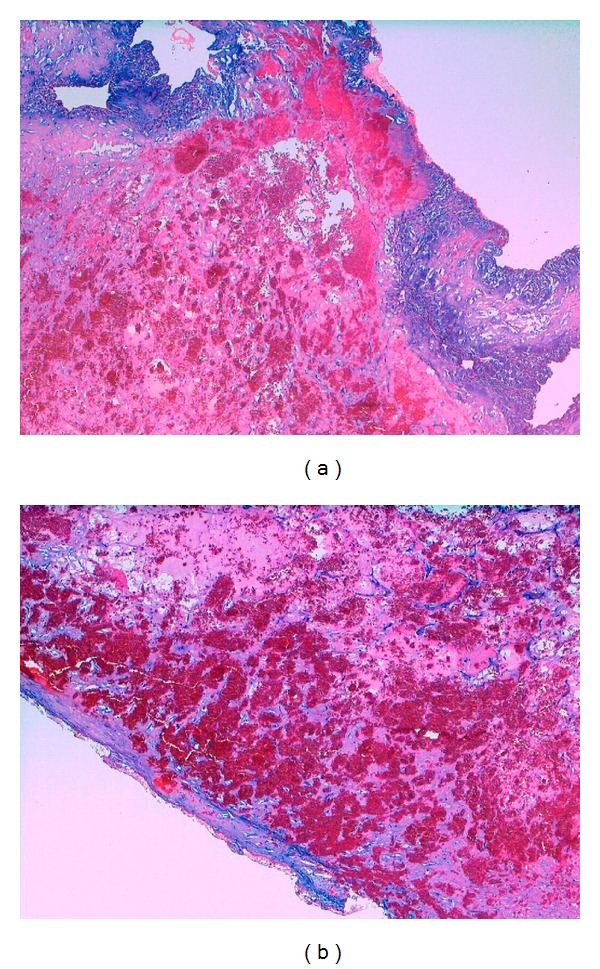
(a) and (b) Capillary hemangioma with proteoglycan-rich stroma within the fibrous capsule (H&E staining, original magnification ×100).

**Figure 6:**
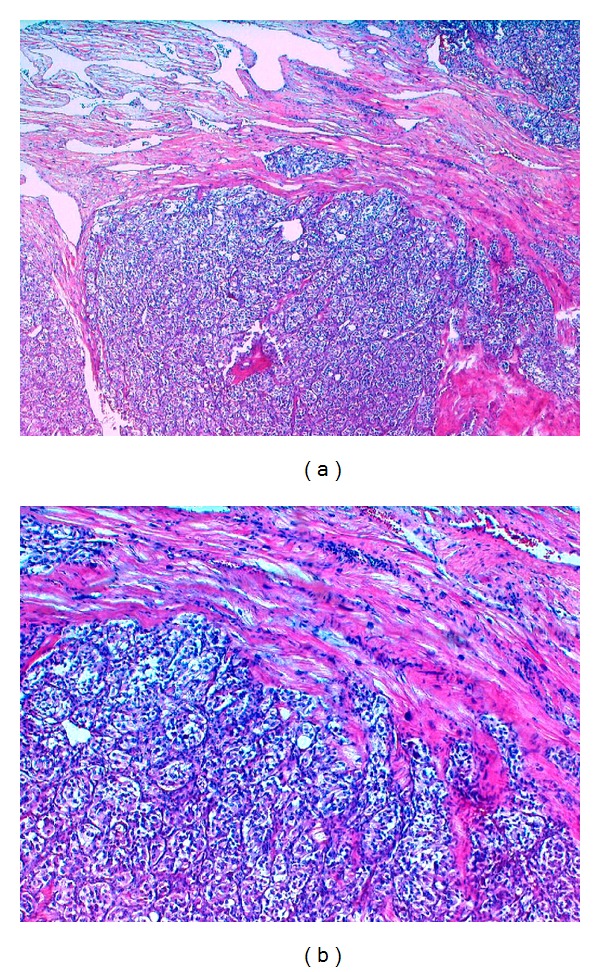
(a) Nests of paraganglioma cells surrounded by a fibrous capsule and adjacent myocardial tissue consisting of myocytes with enlarged nuclei (H&E staining, original magnification ×100); (b) H&E staining, original magnification ×400.

Of a total of 17280 patients receiving heart surgery between January 1970 and December 2011, 78 patients (0.45%) suffered from a heart tumour and received therefore tumourectomy (Table 3). 77 (98.71%) of cardiac tumours were benign, only 1 (1.28%) was malign. 12 patients (15.38%) had a papillary fibroelastoma (Figure 1), 63 patients (80.77%) suffered from myxoma (located in the right or left atrium, Figures 2 and 3), 1 patient (1.49%) had a rhabdomyosarcoma (Figure 4), 1 (1.49%) a hemangioma (Figure 5), and 1 (1.49%) a chemodectoma (Figure 6).

77.78% of the myxoma patients (49) were female and 22.22% (14) were male (versus 33.33% female patients (4) and 66.67% male patients (8) in the case of papillary fibroelastoma). The mean age of cardiac tumour patients at the time of operation was 54.29 ± 13.28 years (range 18 to 83 years). There were no significant differences between the age of patients with myxoma and papillary fibroelastoma (56.00 ± 13.04 years versus 53.50 ± 8.07 years). The mean size of myxomas was much larger than the maximum diameter of papillary fibroelastomas (44.29 ± 21.92 mm versus 11.33 ± 5.87 mm). The recurrence rate was 2.56%. None of the patients underwent heart surgery before or had a family history of myxoma or papillary fibroelastoma. A 61-year-old male patient with a left atrial myxoma developed an intracranial meningioma and adrenal adenoma postoperatively. Another female 47-year-old patient suffering from a left atrial myxoma had a family history of Brugada syndrome.

The majority of papillary fibroelastomas (7) were attached to the aortic valve (5 on the noncoronary leaflet, 1 on the left-coronary leaflet, and 1 on the right-coronary leaflet); one involved the left ventricular septum, one involved the mitral valve, and another one the tricuspid valve. Echocardiographic examination of two patients revealed multiple papillary fibroelastomas (with origin from the left ventricular outflow tract and the aortic valve in one patient and simultaneous occurrence on the aortic and mitral valve in another patient). Histologically, they showed the typical papillar proliferation with branching avascular papillae, anemone-like appearance, and where white coloured with endocardial layer on the surface. 

55 myxomas (87.30%) were located in the left atrium and 8 (12.70%) in the right atrium. 24 myxomas (38.10%) were pedunculated and mobile. Among left atrial myxomas, the major origin was the fossa ovalis (33), followed by the atrial septum (13), the right inferior pulmonary vein (4), the superior pulmonary vein (1), the fornix of the left ventricle (2), the left atrial free wall (1), and in between both pulmonary veins (1). In 16.36% of the cases (9), the tumour mass prolapsed through the mitral valve into the left ventricle leading to mitral valve stenosis and insufficiency, respectively. Right atrial myxomas originated from the right atrial free wall (4) and the right atrial septum (4). One of right atrial myxomas prolapsed through the tricuspid valve into the right ventricle causing tricuspid valve insufficiency.

Histologically, all myxomas were characterized by the myxoma cell, a polygonal or stellate syncytial cell, forming cords or small nests. In 5.56%, additional glandular and pseudocystic elements were found. The proteoglycan-rich myxoid, and vascularized stroma also contained dendritic cells, macrophages, and scattered lymphocytes. Hemorrhage and organizing thrombus resulted in abundant hemosiderin-laden macrophages, which were present in all cases. One myxoma showed Gamna-Gandy bodies, elastic tissue, and calcification surrounding the hemosiderin. Degenerative changes included calcifications in 5.56% and ossifications in 3.70%.

The majority of tumours (58.97%) were found incidentally; 32 patients (41.03%) suffered from symptomatic cardiac tumours including 24 patients (30.77%) with thromboembolic events. 7 patients (8.97%) had clinical symptoms like dyspnoea on exertion, fatigue, stenocardia, or weight loss.

26.87% of the tumour patients (19 patients—4 patients with papillary fibroelastomas and 15 patients with a myxoma) were initially admitted to hospital due to acute cerebral infarction and recurrent transient ischemic attacks, respectively. The majority of ischemic events in the brain (8 patients) affected the middle cerebral artery; MR investigation of 2 patients revealed additional multiple aneurysms of various brain arteries. 9 of the 19 patients (47.37%) suffered from multiple ischemic cerebral events. All myxomas leading to stroke or transient ischemic attacks were localized in the left atrium. 

Among those who did not suffer from cerebral thromboembolic events, 1 patient had pulmonary emboli secondary thrombosis to a leg vein, 1 showed an acute embolic occlusion of the popliteal artery, 1 had an embolic occlusion of the central retinal artery, and other two patients were admitted to hospital due to embolic myocardial infarction.

Recurrent tumours were found in two patients (2.6%). A 52-year-old female patient suffering from a left atrial myxoma developed a recurrent tumour mass 5 months after initial operation. A 20-year-old male patient with a history of weight loss and night sweats had an embryonal rhabdomyosarcoma in the left atrium and underwent surgery in July 1999. Macroscopic examination revealed a white-yellowish tumor with hemorrhage on the surface weighing 110 g and measuring 8 cm in greatest dimension. The histological examination of resected specimens revealed necrosis, spindle-shaped cells with polymorphic nuclei, and numerous mitoses (8 mitoses per high power field). Immunohistochemically, the tumour cells were positive for vimentin, actin, desmin, and myoglobin. Postoperatively, he received doxorubicin-based adjuvant chemotherapy, but metastases right parasternal and on the left upper leg occurred in May 2001 and were resected immediately after diagnosis. Metastases were also found on the right plica vocalis, making a subtotal laryngeal resection and following neck dissection in June 2001 necessary. Afterwards once again 3 cycles of doxorubicin- and cyclophosphamide-based chemotherapy were performed. In January 2005, two rapidly growing metastases in the right cranial hemisphere were found treated by stereotactic radiation therapy. Almost 6 years after initial diagnosis, the tumour recurred. The recurrent tumour mass measuring up to 6 cm in greatest dimension was resected in February 2005. Last followup in 2011 was inconspicuous.

Another 18-year-old man suffered from a tumour mass in the left atrium. He underwent resection in 1999. Intraoperative frozen section was highly suspicious of a rhabdomyosarcoma, but unexpectedly, following histological examination revealed a chemodectoma of the heart, a very uncommon entity. To date, about 40 cases are described in the medical literature. The tumour measured 7 × 7 × 5 cm, consuming the entire left atrium, and showed histologically small nests of eosinophil cells with enlarged nuclei. Immunohistochemically, the tumour cells were positive for chromogranin and CD56, focal positive for S100, and negative for desmin, CD68, actin, SMA, and AE1/AE3. Less than 1% of the tumour cells were positive for Ki67. The postoperative course was uneventful and the tumour did not recur.

## 4. Discussion

The most common heart tumour occurring in our study population is the cardiac myxoma, which constitutes 80% of surgically excised masses. In general, patient age ranges from 2 to 97 years, and the mean age at presentation is 50 years [3]. Our results are in agreement with those who noted a higher percentage of female patients suffering from a myxoma. A recent analysis of multiple surgical series including 1195 individuals with myxomas revealed that 67% were female and 33% were male [4]. Patients with myxoma (Carney) complex are generally younger and more often male in comparison with patients with sporadic myxomas [5, 6]. The clinical presentation is diverse and includes obstructive cardiac symptoms, embolic phenomena, and constitutional symptoms. Embolic myxoma may cause cerebral ischemia, claudication of the extremities, renal failure, coronary obstruction, and sudden death. Most patients have an abnormal physical examination, characteristically with a diastolic or systolic murmur. A “tumour plop” may be occasionally heard in the early diastole. 

In agreement with our results, embolic phenomena are described to occur in 30% to 40% of patients. Frequent sites of embolization include the central nervous system, kidney, spleen, and extremities. Coronary embolism is rare. Smooth-surfaced tumors are more likely to produce valvular obstruction, whereas polypoid and myxoid tumors are more likely to embolize [5]. 

Although we found a higher percentage of incidentally diagnosed myxomas (59%), generally 20% of cardiac myxomas are noted to be asymptomatic; incidental myxomas are usually smaller than 40 mm [7]. Abnormal but nonspecific ECG changes may be identified in 20% to 40% of patients and include atrial fibrillation or flutter and left and right bundle branch block [3]. At echocardiography, cardiac myxomas typically appear as a mobile mass attached to the endocardial surface by a stalk, usually arising from the fossa ovalis. 

We found almost 17% of left atrial myxomas prolapsing through the mitral valve. It is reported that in more than 50% of patients, left atrial myxomas cause symptoms of mitral valve stenosis or obstruction (dyspnea and orthopnea from pulmonary edema or heart failure). Right atrial myxomas may obstruct the tricuspid valve and cause symptoms of right-sided heart failure [3]. 

Myxomas are observed to arise from the endocardium of the left atrial septum near the fossa ovalis in 85% to 90% of cases. Most of the remainder are located in the right atrium. Although most myxomas are sporadic, fewer than 5% of them form a component of the myxoma complex [3]. This autosomal dominant syndrome, known as Carney syndrome, includes cardiac myxomas and extracardiac manifestations: abnormal skin pigmentation (lentigines and blue nevi), calcifying Sertoli-Leydig testicular tumours, cutaneous myxomas, myxoid breast fibroadenomas, pigmented adrenal cortical hyperplasia, pituitary hyperactivity, psammomatous melanotic schwannomas, and thyroid tumours. 

Cause of the high risk for embolization tumour resection is indicated in cases of large or mobile myxomas. The first successful removal of a cardiac myxoma was performed in Stockholm by Clarence Crawford on July 16, 1954 [8]. The patient was the second in the world to survive pulmonary bypass.

Commonly, patients with sporadic tumours have a good prognosis, with a 1% recurrence rate. However, about 10% of patients with familial myxomas either have recurrent tumors or develop another tumour in a different location [5, 6, 9]. Because embolization is the major complication of myxomas, especially of myxoid, fiable, familial tumours, identification of first-degree relatives of patients with documented myxoma syndrome is important. Intracranial aneurysm resulting from embolization is also a rare, but potentially serious complication. The origin of these aneurysms is unclear, but histologic verification of myxoma cells in arterial walls has been reported [10].

Papillary fibroelastomas, also known as fibroelastic papilloma, the most common primary tumour of cardiac valves, occur exclusively on endocardial surfaces, mostly on leaflets. Historically, papillary fibroelastoma was an incidental autopsy finding, seemed to have no clinical significance, and indeed, most papillary fibroelastomas do not cause any symptoms and are usually incidental findings by routine echocardiography. However, early diagnosis of this condition is important, since it represents a surgically correctable cause of systemic emboli, stroke, myocardial infarction, and sudden cardiac death.

Although we observed more male fibroelastoma patients, no gender predominance is reported, and there is a wide range of age at presentation, with a mean age of approximately 60 years [11]. The histogenesis is still a matter of controversial discussion. Papillary fibroelastoma occasionally occurs in areas of previous endocardial damage or in patients with preexisting heart disease. Although none of our heart tumour patients underwent heart surgery before, there is increasing evidence that at least a subset (18%) of cardiac papillary fibroelastomas develop as a result of iatrogenic factors, including thoracic irradiation and open-heart surgery (subaortic septal myectomy, valve repair, valve replacement, and repair of congenital defects) [12]. Most symptoms arise from left-sided lesions that shower fibrin clots into the cerebral circulation or prolapse into the coronary orifice. The most common symptoms are transient neurologic defects, myocardial ischemia [13], and, rarely, sudden cardiac death. Papillary fibroelastomas of the cardiac valves demonstrate typical echocardiographic features [14].

Histologically, they are avascular papillary structures lined by endocardial cells. They are sometimes mistaken for cardiac myxomas, which are vascular and of heterogeneous cell types. Papillary fibroelastomas are treated curatively by surgery, whether there are preexisting embolic symptoms or a lesion is incidentally discovered. Asymptomatic patients can be treated surgically if the tumour is mobile, because the tumour mobility is the independent predictor of death or nonfatal embolization. Asymptomatic patients with nonmobile lesions can be followed up closely with periodic clinical evaluation and echocardiography, and they can receive surgical intervention when symptoms develop or the tumour becomes mobile [11]. Recurrences are rare, and valve-sparing surgery should be considered whenever possible, because partially resected lesions do not always regrow [15]. Papillary fibroelastomas range in size from 2 to 50 mm in greatest dimension, are generally white coloured, and are usually attached to the endocardial surface by a short single stalk. They most often occur singly but multiple tumours (2 to greater than 40) have been described.

In conclusion, cardiogenic embolism due to heart tumours is recognized increasingly as an important cause of stroke, accounting of 20% of ischemic strokes. Cardioembolic stroke is largely preventable. The likelihood of recurrence is relatively high for most cardioembolic sources and therefore secondary stroke prevention is fundamental. The first-choice treatment of symptomatic and mobile cardiac papillary fibroelastomas and cardiac myxomas is surgical excision, which must be performed as early as possible to reduce the risk of early recurrences of embolic events.

## Figures and Tables

**Table 1:** WHO classification of tumours of the heart [2].

Benign tumours and tumour-like lesions
Rhabdomyoma
Histiocytoid cardiomyopathy
Hamartoma of mature cardiac myocytes
Adult cellular rhabdomyoma
Cardiac myxoma
Papillary fibroelastoma
Haemangioma
Cardiac fibroma
Inflammatory myofibroblastic tumor
Lipoma
Cystic tumour of the atrioventricular node
Malignant tumours
Angiosarcoma
Epithelioid hemangioendothelioma
Malignant pleomorphic fibrous histiocytoma
(MFH)/undifferentiated pleomorphic sarcoma
Fibrosarcoma and myxoid fibrosarcoma
Rhabdomyosarcoma
Leiomyosarcoma
Synovial sarcoma
Liposarcoma
Cardiac lymphomas
Metastatic tumours
Pericardial tumours
Solitary fibrous tumour
Malignant mesothelioma
Germ cell tumours
Metastatic pericardial tumours

**Table 2:** Entity and localisation of heart tumours resected between January 1970 and December 2011 at the Medical University of Innsbruck.

Myxoma	63
Left atrium	55
Right atrium	8
Papillary fibroelastoma	12
Aortic valve	7
Mitral valve	1
Aortic and mitral valve	1
Left ventricular septum	1
Aortic valve and left ventricular outflow tract	1
Tricuspid valve	1
Rhabdomyosarcoma	1
Hemangioma	1
Chemodectoma	1

**Table 3:** Annual distribution of heart tumor resections between January 1970 and December 2011.

	Myxoma	Papillary fibroelastoma	Rhabdomyosarcoma	Chemodectoma	Hemangioma
1970–1979	5	0			
1980–1989	2	0			
1990–1999	12	0	1		
2000–2011	35	12		1	1
	**63**	**12**	**1**	**1**	**1**
